# Everything’s Relative? Relative Differences in Processing Fluency and the Effects on Liking

**DOI:** 10.1371/journal.pone.0135944

**Published:** 2015-08-19

**Authors:** Michael Forster, Gernot Gerger, Helmut Leder

**Affiliations:** Department of Basic Psychological Research and Research Methods, Faculty of Psychology, University of Vienna, Vienna, Austria; Center for Music in the Brain (MIB), DENMARK

## Abstract

Explanations of aesthetic pleasure based on processing fluency have shown that ease-of-processing fosters liking. What is less clear, however, is how processing fluency arises. Does it arise from a relative comparison among the stimuli presented in the experiment? Or does it arise from a comparison to an internal reference or standard? To address these questions, we conducted two experiments in which two ease-of-processing manipulations were applied: either (1) within-participants, where relative comparisons among stimuli varying in processing ease were possible, or (2) between-participants, where no relative comparisons were possible. In total, 97 participants viewed simple line drawings with high or low visual clarity, presented at four different presentation durations, and rated for felt fluency, liking, and certainty. Our results show that the manipulation of visual clarity led to differences in felt fluency and certainty regardless of being manipulated within- or between-participants. However, liking ratings were only affected when ease-of-processing was manipulated within-participants. Thus, feelings of fluency do not depend on the nature of the reference. On the other hand, participants liked fluent stimuli more only when there were other stimuli varying in ease-of-processing. Thus, relative differences in fluency seem to be crucial for liking judgments.

## Introduction

The relationship between ease-of-processing and liking is an important issue in theories of processing fluency: the easier a stimulus can be processed the more it is liked [1]. The theory suggests that processing ease—i.e. processing fluency—signals a positive state of affairs, absence of threat, and safety, which are hedonically positive [2]. This positivity is then interpreted as a positive reaction towards the stimulus, resulting in liking. Various studies have shown that higher ease-of-processing indeed is associated with higher liking judgments (see [3], for a review).

What is less clear, however, is how processing fluency arises. Does it arise from a comparison between the experienced processing ease of a stimulus and processing ease of previously seen stimuli? That is, do participants infer processing ease in a relative manner? This would be in accordance with studies showing that evaluations depend on relative differences among stimuli of a given stimulus set [4–6]. Alternatively, one could assume that individuals experience processing fluency of a stimulus with regard to an internal reference or standard in an absolute manner—as for example shown in studies of absolute pitch [7]. In two experiments, we shed light on these questions by comparing effects of ease-of-processing manipulated between-participants or within-participants. This allowed us to study whether the presence of an external reference frame in terms of more or less fluent trials—in a within-participants design—is necessary for processing fluency and for fluency effects on evaluations, such as liking judgments. The experiments compare the two alternatives, and also provide evidence regarding appropriate future research designs for study of fluency-liking effects.

Studying the source of processing fluency is in the tradition of the longstanding question whether a reaction to a stimulus is due to stimulus properties (objectivist, bottom-up), perceiver properties (subjectivist, top-down) or an interaction between those two (interactionist, see [1]). In their seminal article about processing fluency and aesthetic pleasure, Reber and colleagues favour an interactionist account [1]. Pleasure is derived from higher ease-of-processing which arises from an interaction between a perceiver and a stimulus. How this interaction works in detail, however, is not fully understood.

One possible explanation for the origin of processing fluency can be derived from another theory explaining the concept: the discrepancy-attribution hypothesis [8–10]. This hypothesis addresses the effects of processing fluency on feelings of familiarity. The main tenet is that individuals are able to monitor the coherence of their processing. That is, when the experience of processing ease is higher than expected, this discrepancy triggers an attribution process [9]. When ease is attributed to prior experience, feelings of familiarity can arise. Importantly, for processing ease, this model suggests an internal reference frame of how easy something can be processed. This reference frame is then used to compare the local processing experience. The exact manner of how this reference frame is implemented in the cognitive system has not been specified.

In memory research, in which the discrepancy-attribution hypotheses originates, an insight into the ease of retrieval of memory traces—how easy a stimulus comes to mind by comparison to an internal reference frame—seems plausible (see [11]). However, in the case of perceptual fluency [12], ease-of-processing stems from sources such as variations in visual clarity, contrast, or presentation duration. In these cases, it seems more plausible that processing fluency arises through comparisons with an external reference frame. Fluency of a stimulus is higher because it is clearer, has a higher contrast or is perceived longer; but only in relation to other stimuli in the set. This means that the ease in processing a present stimulus is compared with the ease in processing the previous stimuli. Analysing order effects of fluency, Hansen, Dechêne and Wänke ([13], see also [14]) showed that effects of ease-of-processing on truth judgments are strongest in trials when the ease in a trial was discrepant with the previous trial. This suggests that ease-of-processing might be determined relative to previous experiences of ease. However, especially for liking judgments, the importance of other stimuli as a reference for ease-of-processing still remains to be systematically tested.

One possible method to test whether processing fluency is inferred in a relative or absolute manner is to employ a fluency manipulation both within a between-participants and a within-participants design. In a between-participants design, where one group of participants receives easy-to-process stimuli while the other group receives hard-to-process stimuli, relative comparisons would not be possible. However, processing fluency still could be inferred with regard to an internal reference frame. That is, we may still determine whether the processing ease of the stimulus is different from ones’ expectations [8–10]. It is an open question whether such fluency effects due to an internal reference frame (in between-participants manipulations of ease-of-processing) are similar to fluency effects due to an external reference frame (in within-participants manipulations).

To our knowledge, in processing fluency research only one study by Lanska, Olds, and Westerman [15] used both between-participants and within-participants manipulations of processing ease. Though the experiments were designed to compare effects of perceptual and conceptual fluency on recognition judgments, in Experiment 1a fluency was manipulated between-participants and in Experiment 1b fluency was manipulated within-participants. The effects of both perceptual and conceptual fluency on recognition judgments were similar regardless whether these were tested between- or within-participants. Thus, in the between-participants design used in this study, participants presumably employed an internal reference frame, which led to a consistent experience of fluency. This is also in line with the discrepancy-attribution hypothesis, which predicts use of an internal reference for feelings of familiarity. However, for perceptual fluency manipulations and for liking judgments, this may not be the case.

Thus, given the sparse empirical data, we conducted two experiments that differed in regards to whether an ease-of-processing manipulation varied between-participants or within-participants. To manipulate ease-of-processing, simple line drawings were either presented with overlaid random noise (low visual clarity and low ease-of-processing) or without random noise (high visual clarity, high ease-of-processing). In addition to this manipulation, we presented the stimuli at different presentation durations (within-participants, see [12, 16, 17]). Therefore, in Experiment 1, visual clarity served as a between-participants manipulation of ease-of-processing with an additional within-participants manipulation of ease-of-processing through presentation duration. In Experiment 2, both visual clarity and presentation duration were manipulated within-participants (see Table 1).

**Table 1:** Experimental design of Experiments 1 and 2.

	Ease-of-processing manipulation	
	Presentation Duration (100, 200, 300, 400 ms)	Visual Clarity (noise, no-noise)
**Experiment 1 (n = 64)**	Within-participants	Between-participants
		no-noise condition: n = 32, noise condition: n = 32
**Experiment 2 (n = 33)**	Within-participants	Within-participants
	

This procedure was chosen because using two manipulations of perceptual fluency allows us to study joint effects on ease-of-processing. This also more closely resembles our everyday experiences where sources of fluency are manifold (e.g., presentation duration, visual clarity, and repetition, see [18]).

In both experiments, we tested the effects of the manipulations on three main variables: felt fluency, liking, and certainty. Measures of felt fluency indicated whether both manipulations indeed lead to a subjective experience of fluency [19, 20]. It is an ongoing debate whether processing fluency itself can be consciously reported. Reber, Fazendeiro, and Winkielman [21] argue that processing fluency “is typically experienced at the periphery of the conscious awareness, resulting in a vague or ‘fringe’ experience of ease” (p. 3). However, recent findings indicate that participants do have conscious insight into their ease-of-processing [16, 19, 20]. When higher processing ease is only reflected in higher liking [21], or in shorter reaction times ([19], but see [22]), then our manipulations might not be reflected in felt fluency. However, irrespective of conscious insight into processing ease, higher ease-of-processing should be reflected in higher liking ratings. Therefore, we tested both felt fluency and liking. With the additional measure of certainty—how certain are participants about their liking decision—we tried to capture another facet of fluency. According to Reber, Schwarz, and Winkielman, higher ease-of-processing signals “progress toward successful recognition of the stimulus, error-free processing, or the availability of appropriate knowledge structures to interpret the stimulus” ([1], p.366, see also [2]). If fluency indicates successful recognition or error-free processing, then participants might be more confident about their perception, resulting in higher certainty judgments (see also [23, 24]).

## Experiment 1

### Method

#### Ethics Statement

Both experiments were conducted in accordance with the Declaration of Helsinki (revised, 1983) and the guidelines of the Faculty of Psychology, University of Vienna. According to the Austrian Universities Act 2002 (UG2002) which was active at the time the study was carried out, only medical universities were required to appoint ethics committees for clinical testing, application of medical methods and applied medical research. Therefore, no ethical approval was sought for the experiments. All participants provided written informed consent prior to their participation. Participants were informed that participation and data collection was fully anonymous and that they could withdraw at any time during the experiment without any further consequences. Each participant received an anonymized code upon beginning the study which was used to identify both consent form and the results sheet containing main study results. The pre-screening for eyesight and color vision was administered by the experimenter (MF), with results also recorded on a separate anonymized sheet. Consent forms were stored separately from the data sheet, with the former (physical paper) held in a locked cabinet and the latter (digital records of study answers/reaction times) stored in a password protected server. Thus, only the administering researcher (MF) had access to both age/names and study results. Co-authors had access to the anonymized results only. The consent forms and codes were kept in order to ensure the rights of the participants to later inspect their own data or to have their data deleted upon request.

#### Participants

Sixty-four volunteers (48 women) participated in Experiment 1. Mean age was 22.5 years (*SD* = 4.1, age range = 18–44). In a pre-screening we ensured that all participants had normal vision.

#### Stimuli

**Fig 1:**
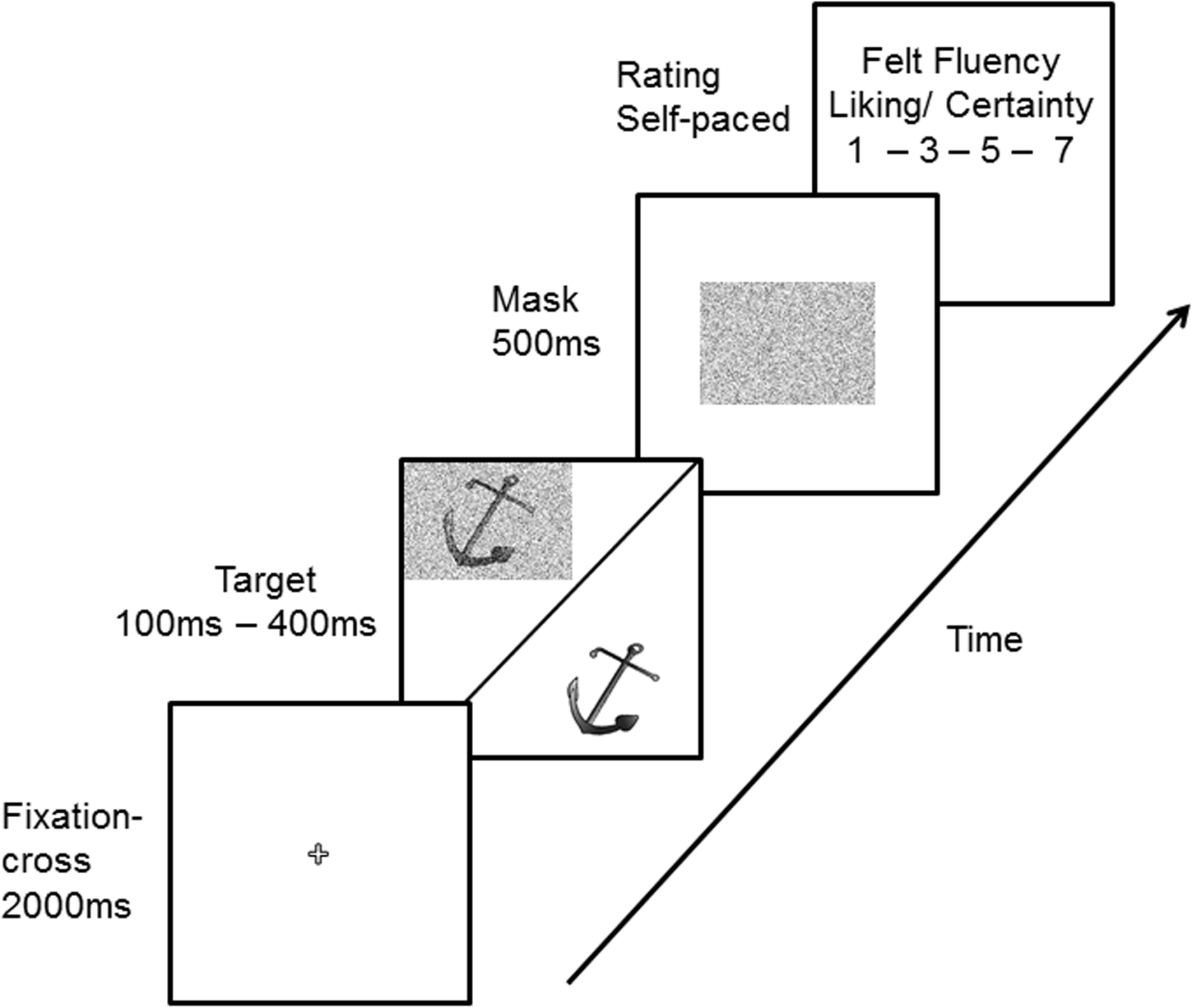
Trial sequence with respective presentation durations to the left. In Experiment 1, half of the participants were presented with targets with Gaussian noise, the other half of the participants were presented with targets without Gaussian noise. In Experiment 2, participants were presented with both types of targets (with and without Gaussian noise).

We selected 96 black-and-white line pictures (image size: 3.2 × 2.3 in., or 6.79° × 4.75° of visual angle at a viewing distance of approx. 27.56 in.) from the picture set of Rossion and Pourtois [25]. This set comprises simple drawings of everyday objects, such as an anchor, dog, spoon etc. For the condition with low visual clarity, and hence low ease-of-processing, we added 60% Gaussian noise to the original picture (see also [12]). To assure that visual processing did not outlast the presentation duration, a masking stimulus consisting solely of 60% Gaussian noise was created (see Fig 1).

#### Design and Procedure

Half of the participants (n = 32) viewed the original images without any image manipulation (no-noise condition, with high visual clarity/ease-of-processing). The other half viewed the images with 60% Gaussian noise added (noise condition, with low visual clarity/ease-of-processing). The experiment consisted of two blocks: (1) one in which all stimuli were rated for felt fluency, and (2) one in which they were rated for liking and certainty. In both blocks, ease-of-processing was additionally manipulated by varying the presentation duration in 100 ms steps between 100 and 400 ms [12, 16]. To avoid repetition of the images, and in order to reduce effects when felt fluency and liking are recognized as belonging together [26], felt fluency was rated in one block and liking as well as certainty in a another block.

The assignment of participants to the noise or no-noise condition was pseudo-randomized upon their arrival in the lab. For each participant the four different presentation durations (100, 200, 300, or 400 ms) were randomly assigned to the 96 stimuli. The only constraint involved assuring that each of the presentation durations occurred equally often (i.e., 24 times). Consequently, for each participant each stimulus was presented in only one of the four presentation durations. The order of the rating blocks (felt fluency or liking/certainty) was counterbalanced across participants. In the second rating block, the stimuli were repeated with the same presentation duration as in the first rating block. The order of the stimuli, however, was randomized.

In order to conceal the link between the felt fluency block and the liking/certainty block, the experiment started with an instruction phase in which the participants were told that there were two independent blocks of ratings to be made. After this instruction, participants were familiarized with the task in four practice trials. Each trial started with a fixation cross for 2 s, followed by the target image for 100, 200, 300 or 400 ms. A subsequent 500 ms noise mask limited visual processing of the target. Finally, depending on block order, the participants gave felt fluency (“How easy was the perception of the presented stimulus?”) or liking (“How much do you like the picture?”) and certainty (“How certain are you in your judgment?”) ratings. All ratings were given on a Likert-type scale. For the felt fluency rating the scale ranged from 1 (very easy) to 7 (very hard). For the liking and certainty ratings the scale ranged from 1 (not at all/very uncertain) to 7 (very much/very certain). For data analysis, the felt fluency rating scale was inverted to simplify interpretation. Higher ratings consequently represent higher felt fluency, liking or certainty. Within blocks, presentation order of stimuli and different durations were randomized. The experiment was run with E-Prime 2.0 [27] and presented on a 19” display at a resolution of 1,280 × 1,024 pixels and a refresh rate of 60 Hz. After completing the experiment, the participants filled out post-questionnaires regarding the assumed purpose of the experiment. Finally, participants were thoroughly debriefed and thanked for their participation.

### Results and Discussion

To test the effect of both ease-of-processing manipulations on the dependent variables, we ran three mixed factors analyses of variance (ANOVA): one for felt fluency, one for liking, and one for certainty. For all three ANOVAs the between-participants factor was stimulus condition (noise or no-noise) and the within-participants factor was presentation duration (100, 200, 300, or 400 ms). In all analyses the level of statistical significance was set at *p* < .05. In cases where sphericity assumption was violated, Greenhouse-Geisser correction was applied. For analyses of the different presentation durations, we performed linear trend analyses, given our hypothesis that ratings of felt fluency, liking, and certainty should linearly increase with longer presentation durations. To further investigate interaction effects we either performed separate ANOVAs for the two stimulus conditions or multiple pairwise comparisons with Bonferroni correction.

#### Felt fluency

**Fig 2:**
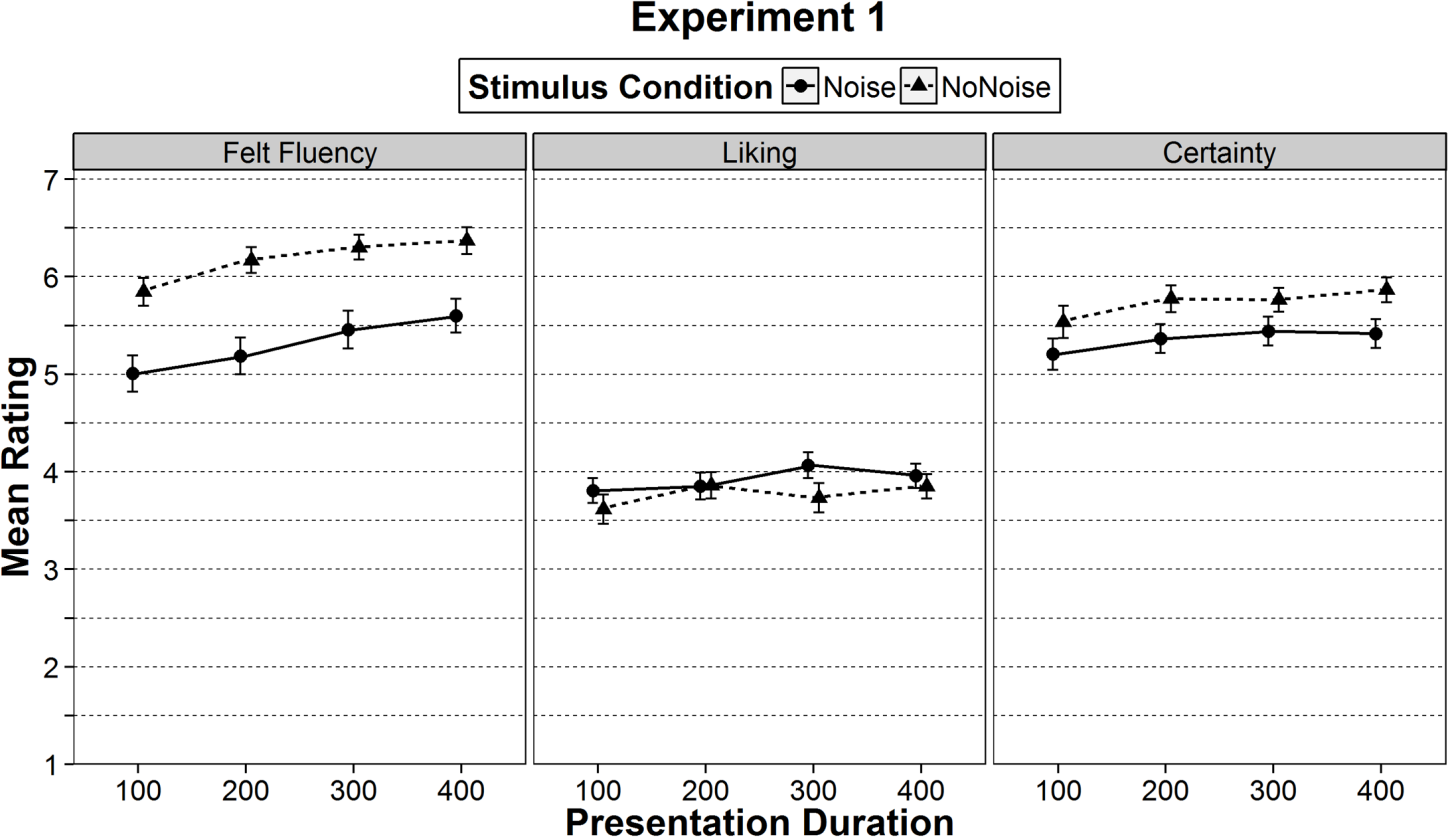
Mean ratings of felt fluency, liking, and certainty in Experiment 1. Means are separated by ease-of-processing (presentation duration of 100, 200, 300, or 400 ms) and by stimulus condition (noise or no-noise). The error bars represent ± one standard error of the mean.

Testing whether both manipulations of ease-of-processing yielded differences in the experience of fluency, ANOVA showed a main effect of stimulus condition, *F*(1, 62) = 15.43, *p* < .001, η_p_^2^ = .20, and a main effect of presentation duration, *F*(2.4, 150.8) = 37.47, *p* < .001, η_p_^2^ = .38. However no interaction was detected, *F*(2.4, 150.8) = 1.23, *p* = .300, η_p_^2^ = .02. Felt fluency was higher in the stimulus condition without noise than in the stimulus condition with noise (*p* < .001, see Table 2 and Fig 2). Linear trend analysis showed that felt fluency significantly increased with presentation duration, *F*(1, 62) = 70.54, *p* < .001, η_p_^2^ = .53. These results showed that differences in ease-of-processing both between and within the participants were reflected in differences in felt fluency. Thus, in the case of visual clarity no external reference was needed for fluency to be experienced. In line with the discrepancy attribution hypothesis [9, 10], participants may have relied on an internal reference frame. The analysis for liking ratings tested whether these differences in felt fluency translated into differences in liking, as suggested by processing fluency approaches to aesthetic pleasure [1].

**Table 2:** Means and standard deviations (in parentheses) for felt fluency, liking, and certainty in Experiment 1.

		Experiment 1			
Dimension	Condition	Ease-of-processing			
		100ms	200ms	300ms	400ms
Felt Fluency	Noise	5.01 (1.05)	5.19 (1.07)	5.46 (1.10)	5.60 (0.98)
	No-Noise	5.85 (0.81)	6.17 (0.74)	6.30 (0.72)	6.37 (0.77)
Liking	Noise	3.80 (0.72)	3.85 (0.79)	4.07 (0.74)	3.96 (0.71)
	No-Noise	3.62 (0.84)	3.86 (0.77)	3.73 (0.86)	3.85 (0.71)
Certainty	Noise	5.21 (0.90)	5.37 (0.84)	5.44 (0.83)	5.42 (0.83)
	No-Noise	5.54 (0.94)	5.77 (0.77)	5.76 (0.68)	5.86 (0.72)

Means are separated by ease-of-processing (presentation duration of 100, 200, 300, or 400 ms), by stimulus condition (noise or no-noise).

#### Liking

For ratings of liking the ANOVA showed no significant main effect of stimulus condition, *F*(1, 62) = 0.75, *p* = .389, η_p_^2^ = .01, but a significant main effect of the within-participants factor presentation duration, *F*(3, 186) = 5.07, *p* = .002, η_p_^2^ = .08. This was also qualified by a significant interaction between stimulus condition and presentation duration, *F*(3, 186) = 3.15, *p* = .026, η_p_^2^ = .05. The interaction was mainly due to the fact that for the group with noise, the linear trend for higher liking with increasing presentation duration was stronger, *F*(1, 31) = 5.12, *p* = .031, η_p_^2^ = .14, than for the group without noise, *F*(1, 31) = 4.09, *p* = .052, η_p_^2^ = .12. Taken together, in both stimulus conditions, liking ratings tended to be higher at longer durations.

Separate comparisons between the stimulus conditions for the four presentation durations yielded no significant differences (*p*s > .101). As evident from Fig 2 (left column), the mean liking ratings in the no-noise condition with high visual clarity—where ease-of-processing was higher—tended to be even lower compared to the noise condition with low visual clarity. This indicated that higher ease-of-processing, when varied between-participants, was not accompanied by higher liking ratings. This finding however could not be attributed to lack of felt fluency difference between the two stimulus conditions. As shown above, felt fluency was influenced by the between-participants manipulation of ease-of-processing. Nonetheless, for the between-participants factor, the differences in felt fluency did not translate into differences in liking. It rather appeared that only the experience of a relative higher fluency of longer presented stimuli—within-participants—results in higher liking [1].

#### Certainty

Following the processing fluency theories, when a stimulus can be processed fluently participants should also be more certain about their evaluations [23, 24]. Thus, participants rated how certain they were about their liking evaluations. ANOVA with certainty ratings as a dependent variable showed a trend of stimulus condition, *F*(1, 62) = 3.71, *p* = .059, η_p_^2^ = .06, as well as a significant main effect of presentation duration, *F*(3, 186) = 12.10, *p* < .001, η_p_^2^ = .16, but no interaction, *F*(3, 186) = 0.80, *p* = .495, η_p_^2^ = .01. As shown in Fig 2, participants in the stimulus condition without noise tended to be more certain than in the condition with noise. Furthermore, certainty ratings increased with presentation duration, *F*(1, 62) = 25.51, *p* < .001, η_p_^2^ = .29. Certainty ratings, therefore, mirrored effects of felt fluency (see also [23, 24, 28]).

#### Order effects

Repeating the stimuli in the two blocks posed the possibility of block order effects. For all three dimensions (felt fluency, liking, and certainty) mixed-design ANOVAs with block order and stimulus condition as between-participants factors and presentation duration as within-participants factor were performed. For the sake of parsimony only main effects of block order and interactions including block order are reported. For felt fluency, the ANOVA showed a trend for an interaction between block order and presentation duration, *F*(2.48, 148.52) = 2.40, *p* = .082, η_p_^2^ = .04. To further inspect the interaction, we performed two ANOVAs separately for the two block orders. As indicated by the effect sizes, there was a stronger linear trend for presentation duration, when felt fluency was tested in the second block, *F*(1, 30) = 59.494, *p* < .001, η_p_^2^ = .67, compared to when felt fluency was tested in the first block, *F*(1, 30) = 20.76, *p* < .001, η_p_^2^ = .41. Importantly, this interaction does not qualify the previous findings. For liking, no effect was significant (*p*s > .157). For certainty, an ANOVA showed a significant main effect of block order, *F*(1, 60) = 4.45, *p* = .039, η_p_^2^ = .07. Certainty ratings were higher when tested in the second block.

## Experiment 2

The results from Experiment 1 indicate that both relative and absolute differences of ease-of-processing are subjectively felt as fluent. Furthermore, these differences were also reflected in certainty ratings. That is higher ease-of-processing accompanied higher certainty in judgments. However, only the differences in relative fluency (i.e. differences in presentation duration) also revealed differences in liking. In both stimulus conditions, longer presentation durations were associated with higher liking. However, images presented without noise were not generally liked more. One possible reason might be that differences in visual clarity (i.e. presence or absence of noise) in general do not translate into differences in liking. This however seems unlikely, as visual clarity in general is associated with higher liking [12, 29] and as differences in stimulus clarity were associated with differences in felt fluency. Nonetheless, to rule out this possibility and to explicitly test whether differences in visual clarity translate into differences in liking when visual clarity is relatively higher, we conducted a similar experiment with the added manipulation of both presentation duration as well as visual clarity within-participants.

### Method

#### Participants

Thirty-three volunteers (17 female) participated in Experiment 2. Mean age was 30.6 years (*SD* = 7.0, age range = 21–49). In a pre-screening we ensured that all participants had normal vision. None of the participants took part in Experiment 1.

#### Stimuli

The same stimuli as in Experiment 1 were used.

#### Design and Procedure

The only difference from Experiment 1 was that stimuli with and without noise were presented within participants. That is, participants were presented with stimuli both with high and low visual clarity. For each participant the four different presentation durations (100, 200, 300, or 400 ms) and the two stimulus conditions (noise or no-noise) were randomly assigned to the 96 stimuli. Each of the presentation durations in combination with the two stimulus conditions were however presented equally often (12 times). Consequently, for each participant, each stimulus was presented in only one of the four presentation durations and either with or without noise. All other aspects were identical to Experiment 1.

### Results and Discussion

We ran three repeated measures analyses of variance (ANOVA): one for felt fluency, one for liking, and one for certainty. For all three ANOVAs, the within-participants factors were stimulus condition (noise or no-noise) and presentation duration (100, 200, 300, or 400 ms).

#### Felt fluency

**Fig 3:**
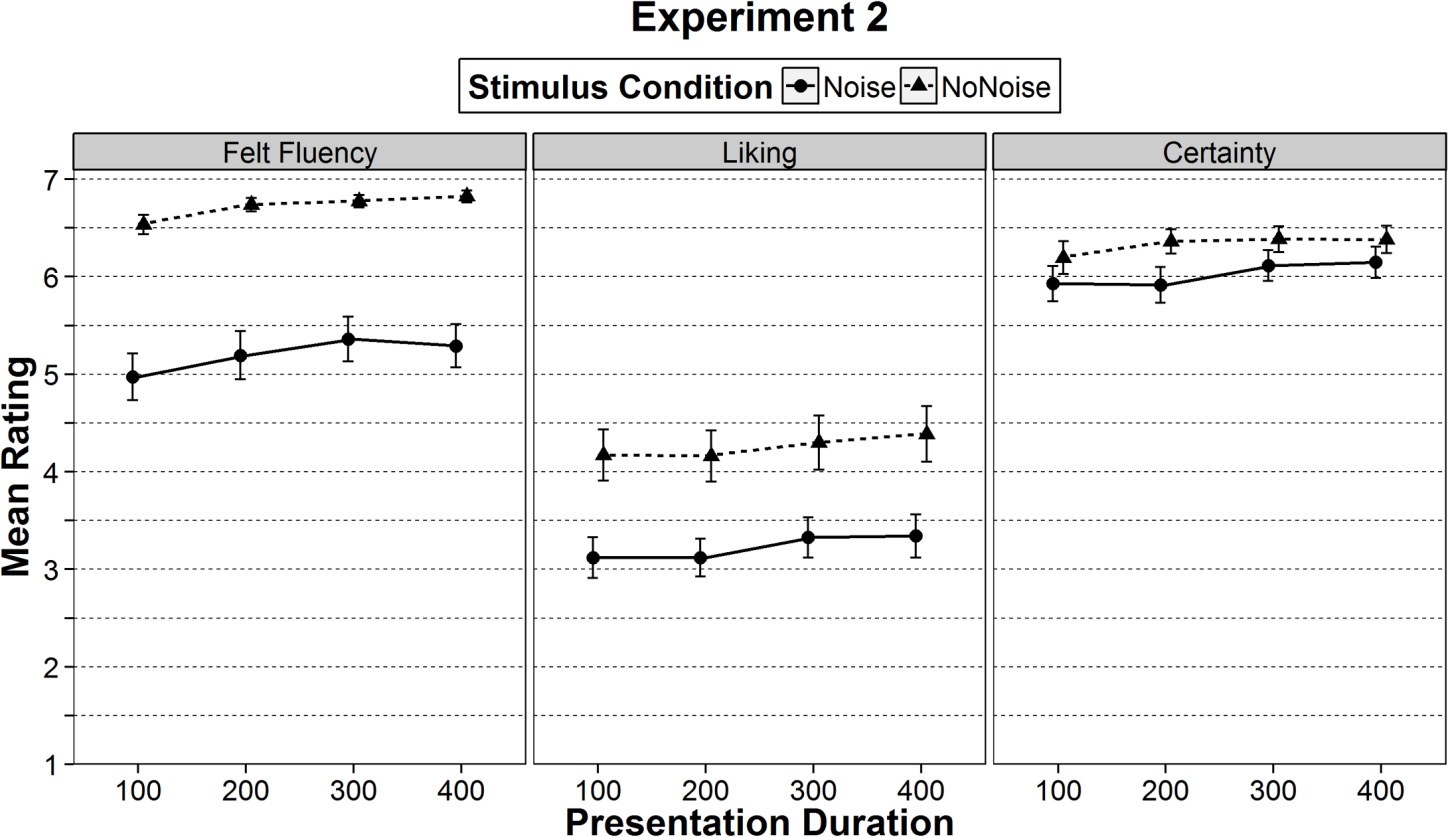
Mean ratings of felt fluency, liking, and certainty in Experiment 2. Means are separated by ease-of-processing (presentation duration of 100, 200, 300, or 400 ms) and by stimulus condition (noise or no-noise). The error bars represent ± one standard error of the mean.

The ANOVA showed a main effect of stimulus condition, *F*(1, 32) = 64.99, *p* < .001, η_p_^2^ = .67, a main effect of presentation duration, *F*(3, 96) = 11.74, *p* < .001, η_p_^2^ = .27, but no interaction, *F*(3, 96) = 0.56, *p* = .641, η_p_^2^ = .02. Felt fluency significantly increased with presentation duration, *F*(1, 32) = 20.44, *p* < .001, η_p_^2^ = .39 (see also Fig 3). Felt fluency was also rated higher for stimuli without noise than for stimuli with noise (see Table 3 and Fig 3). This result showed that differences in ease-of-processing of both manipulations were reflected in differences in felt fluency. Manipulations of presentation duration and stimulus noise were also additive. As indicated by the high means for the stimuli without noise (dashed line in the left column in Fig 3), the ratings of felt fluency appeared to reach a ceiling. As indicated by the effect sizes, the ease-of-processing differences through visual clarity (noise or no-noise) led to stronger differences in felt fluency than the ease-of-processing differences through presentation duration. Thus, when both manipulations were presented within-participants, visual clarity was a more powerful manipulation of ease-of-processing.

**Table 3:** Means and standard deviations (in parentheses) for felt fluency, liking, and certainty in Experiment 2.

Experiment 2					
Dimension	Condition	Ease-of-processing			
		100ms	200ms	300ms	400ms
Felt Fluency	Noise	4.97 (1.37)	5.19 (1.41)	5.36 (1.31)	5.29 (1.28)
	No-Noise	6.54 (0.58)	6.74 (0.39)	6.78 (0.36)	6.82 (0.35)
Liking	Noise	3.12 (1.20)	3.12 (1.51)	3.33 (1.19)	3.34 (1.27)
	No-Noise	4.17 (1.51)	4.16 (1.50)	4.30 (1.60)	4.39 (1.63)
Certainty	Noise	5.93 (1.04)	5.92 (1.06)	6.12 (0.90)	6.15 (0.92)
	No-Noise	6.19 (0.97)	6.36 (0.71)	6.38 (0.76)	6.38 (0.80)

Means are separated by ease-of-processing (presentation duration of 100, 200, 300, or 400 ms), by stimulus condition (noise or no-noise).

#### Liking

For ratings of liking, the ANOVA showed a significant main effect of stimulus condition, *F*(1, 32) = 14.15, *p* < .001, η_p_^2^ = .31, a significant main effect of presentation duration, *F*(3, 96) = 3.72, *p* = .014, η_p_^2^ = .10, but no significant interaction between stimulus condition and presentation duration, *F*(3, 96) = 0.14, *p* = .939, η_p_^2^ < .01. As indicated in Fig 3, stimuli without noise, which are easier to perceive, were liked more. Liking also significantly increased with presentation duration, *F*(1, 32) = 8.61, *p* = .006, η_p_^2^ = .21. In contrast to Experiment 1, both manipulations of fluency exerted their influence on liking. As indicated by the effect sizes, the manipulation of visual clarity did so more strongly (η_p_^2^ = .31) than the manipulation of presentation duration (η_p_^2^ = .10). Taken together, this finding strengthens the argument that the experience of a relative higher fluency—within-participants—is necessary for increases in liking due to ease-of-processing.

#### Certainty

An ANOVA on certainty ratings showed a significant main effect of stimulus condition, *F*(1, 32) = 7.21, *p* = .011, η_p_^2^ = .18, a significant main effect of presentation duration, *F*(2.4, 77.6) = 7.53, *p* < .001, η_p_^2^ = .19, but no interaction, *F*(1.8, 56.5) = 1.25, *p* = .291, η_p_^2^ = .04. As indicated in Fig 3, participants in the stimulus condition without noise were more certain than in the condition with noise (*p* = .011). Furthermore, certainty ratings increased with presentation duration, *F*(1, 32) = 15.74, *p* < .001, η_p_^2^ = .33. The similarity between certainty ratings and felt fluency ratings indicated that certainty might indeed be an alternative measure of processing fluency.

#### Order effects

For felt fluency, a mixed-design ANOVA with block order as a between-participants factor and stimulus condition and presentation duration as within-participants factors showed a significant main effect of block order, *F*(1, 31) = 5.21, *p* = .029, η_p_^2^ = .14, qualified by a significant interaction between block order and stimulus condition, *F*(1, 31) = 4.28, *p* = .047, η_p_^2^ = .12. There also was a trend for an interaction between block order and presentation duration, *F*(2.42, 75.11) = 2.41, *p* = .086, η_p_^2^ = .07. When felt fluency was tested first, ratings were higher than when tested second. The interaction effects are mainly due to the fact that the differences between first and second block were only significant in the noise condition (*p* = .031; no-noise, *p* = .097). The trend for the interaction with presentation duration was due to the difference between first and second block not being significant in the 300 ms condition (*p* = .078, all other *p*s < .048). These interactions do not qualify the previous findings. For liking and certainty no effect, including block order, was significant (*p*s > .146).

## General Discussion

In two experiments we tested whether an external reference frame is needed for fluency effects, or whether fluency also arises without this. Our results show that manipulations of ease-of-processing lead to higher feelings of fluency and higher certainty, regardless of whether other stimuli vary in ease-of-processing or not. However, effects of higher ease-of-processing on liking were only found when participants could experience relative differences in ease-of-processing. In the following we consider this, on the surface rather inconsistent, finding.

For felt fluency effects of within-participants manipulations (Experiment 1: presentation duration, Experiment 2: visual clarity, presentation duration), our results are in line with previous research on processing fluency (e.g., [12, 16]). Thus, the explanation is straightforward: Participants could compare the felt fluency in each trial with the felt fluency of other trials. If a stimulus was presented longer than another stimulus, it felt more fluent. This is quite consistently shown in the ratings (see left columns in Figs 2 and 3). For the manipulation of visual clarity in Experiment 2 the same logic can be applied. If a stimulus was higher in visual clarity than another stimulus, it felt more fluent.

However, felt fluency ratings for visual clarity also differed in Experiment 1, where participants could not perform a comparison between high and low visual clarity. These findings, importantly, show that feelings of felt fluency can arise without experiencing relative differences in ease-of-processing among stimuli. This is in line with Whittlesea and Williams [9, 10], who suggest that fluency can also arise from a comparison between experienced and expected ease. This further suggests that for manipulations of perceptual fluency individuals can apply an internal reference frame regarding how easy something can be processed. To our knowledge this is the first time an external and an internal reference frame in processing fluency have been directly compared.

Though previous experiments have shown that differences in ease-of-processing can be reported by participants [16, 19, 20], one might still argue that ratings of felt fluency are an improper measure of ease-of-processing. We thus measured certainty as an additional measure of ease-of-processing [23, 24]. The results for certainty indicated that easier to process stimuli lead to a higher certainty in liking ratings. Thus, higher ease-of-processing might accompany participants also being more confident that they have successfully perceived the stimulus itself [2, 23, 24]. Consequently, this confidence might have been used as a cue for the certainty in liking ratings.

Taken together, manipulating the ease-of-processing through visual clarity led to differences in felt fluency in both experiments. These results indicate that felt fluency can be inferred relative to an external reference (i.e., compared to other stimuli) and relative to an internal reference (i.e., an internal standard). Whether these feelings of fluency stemming from an external or internal reference reflect a similar quality or a similar internal process cannot be inferred from our results. However, according to Alter and Oppenheimer [3] such a distinction might not be important, as fluency can arise from a variety of sources but may result in very similar outcomes. This is also shown in our study. Regardless of the context of manipulation—between or within—or the type of manipulation—visual clarity or presentation duration—certainty and felt fluency ratings increased with ease-of-processing.

Liking ratings, however, were differentially affected by the ease-of-processing manipulations. At this point we can mainly rule out explanations of why liking judgments were not affected when variations in ease-of-processing due to visual clarity were manipulated between-participants (Experiment 1). First, it can be ruled out that the between-participants manipulation in visual clarity failed. The effects on felt fluency and certainty are indicative of its success.

Second, discounting of fluency—a concept often discussed in processing fluency research [26]—can also be ruled out. Discounting means that when participants become aware of the manipulation of ease-of-processing, they disregard feelings of fluency as a source for their liking ratings. Accordingly, within-participants compared to between-participants manipulations should be more prone to discounting effects as manipulations are more apparent in the within-participants condition. Thus, particularly liking ratings in the within-participants manipulations should have suffered from discounting. This was not the case. Furthermore, for ratings of liking the awareness of the manipulation was certainly higher in participants who received the liking block second. Following the discounting argument these participants should show weaker effects of ease-of-processing on liking. As indicated by the absence of any effect of block order on liking in Experiments 1 and 2, this was not the case. Taken together we could find no evidence for discounting in our experiments.

One possibility why felt fluency did not affect liking in the between-participants condition is that it was not regarded as a valid source for liking ratings. In the absence of an external comparison for visual clarity, the salience of the fluency feeling may not have been high enough to be applied as a source of information (see also [30]). The inspection of the effect sizes supports this hypothesis. In Experiment 1, effect sizes indicate a stronger felt fluency effect for presentation duration (within-participants) compared to visual clarity (between-participants). Thus, the presentation duration manipulation might have been more salient. In Experiment 2 however, effect sizes indicated a stronger felt fluency effect for visual clarity (within-participants) compared to presentation duration (within-participants). This difference in felt fluency strength is also reflected in a stronger effect size in the liking ratings for visual clarity. Thus in Experiment 1, not visual clarity [29] but other features of the stimuli, such as roundness [31], symmetry [32] or personal taste, might have governed the liking ratings. This possibility however requires further testing.

One shortcoming of the measurements should also not go unnoticed. As indicated by the high means in Experiment 2, certainty and felt fluency ratings at the highest levels of ease-of-processing nearly reached a ceiling. The means between the 300 and 400 ms presentation durations for stimuli of high visual clarity did not further increase. This indicates that felt fluency, and hence liking, may potentially not be increased indefinitely. Thus, with stimuli with high visual clarity these presentation durations do not elicit any stronger feelings of fluency or certainty. We used the variations between 100 and 400 ms because we first wanted to follow presentation durations previously used in processing fluency research [12, 16]. Second, we wanted to ensure that stimuli at the lowest levels of ease-of-processing (short presentation duration of stimuli with noise) were identifiable. Depending on stimulus category, in presentation durations below 100 ms identifiability drops considerably [33]. Thus, when presenting stimuli shorter than 100 ms, liking differences might be due to some stimuli being visible and others not. However, in our study, informal questions after the experiment did indicate that participants were able to identify the depicted objects. To allow an optimal discrimination in felt fluency among all levels of manipulations, futures studies nonetheless should avoid making the processing of stimuli too easy.

To sum up, we could show that feelings of fluency arise from both within-participants and between-participants manipulations of ease-of-processing. Thus, it seems that individuals can judge felt fluency both in relation to an external reference and in relation to an internal reference. We can only speculate whether this effect will also be found with other more complex or more ecologically valid stimuli. It might be that, with faces or consumer products the effects of other types of fluency (for example previous experience or familiarity), and other sources for liking (private taste or attitudes) override the subtle effect of perceptual fluency. What our experiments, to our knowledge for the first time, show is that effects of fluency on liking depend on the presence of relative differences in felt fluency. Participants like fluent stimuli more only when there are other stimuli varying in ease-of-processing present.

## Supporting Information

S1 File Data-sheets from Experiments 1 and 2. (ZIP)
